# Non-invasive imaging of tau-targeted probe uptake by whole brain multi-spectral optoacoustic tomography

**DOI:** 10.1007/s00259-022-05708-w

**Published:** 2022-02-07

**Authors:** Patrick Vagenknecht, Artur Luzgin, Maiko Ono, Bin Ji, Makoto Higuchi, Daniela Noain, Cinzia A. Maschio, Jens Sobek, Zhenyue Chen, Uwe Konietzko, Juan A. Gerez, Roland Riek, Daniel Razansky, Jan Klohs, Roger M. Nitsch, Xose Luis Dean-Ben, Ruiqing Ni

**Affiliations:** 1 Institute for Regenerative Medicine, University of Zurich, Zurich, Switzerland; 2 Zentrum für Neurowissenschaften Zürich (ZNZ), Zurich, Switzerland; 3 Institute for Biomedical Engineering and Institute of Pharmacology and Toxicology, Faculty of Medicine, ETH Zurich & University of Zurich, Zurich, Switzerland; 4 National Institutes for Quantum and Radiological Science and Technology, Chiba, Japan; 5 Department of Radiopharmacy and Molecular Imaging, School of Pharmacy, Fudan University, Shanghai, China; 6 Neurology Department, University Hospital Zurich, Zurich, Switzerland; 7 Functional Genomics Center, University of Zurich, Zurich, Switzerland; 8 Laboratory of Physical Chemistry, Department of Chemistry and Applied Biosciences, ETH Zurich, Zurich, Switzerland

**Keywords:** Animal model, Fluorescence imaging, Frontotemporal dementia, Optoacoustic imaging, Tau

## Abstract

**Purpose:**

Abnormal tau accumulation within the brain plays an important role in tauopathies such as Alzheimer’s disease and frontotemporal dementia. High-resolution imaging of tau deposits at the whole-brain scale in animal disease models is highly desired.

**Methods:**

We approached this challenge by non-invasively imaging the brains of P301L mice of 4-repeat tau with concurrent volumetric multi-spectral optoacoustic tomography (vMSOT) at ~ 115 μm spatial resolution using the tau-targeted pyridinyl-butadienyl-benzothiazole derivative PBB5 (*i.v.*). In vitro probe characterization, concurrent vMSOT and epi-fluorescence imaging of in vivo PBB5 targeting (*i.v.*) was performed in P301L and wild-type mice, followed by ex vivo validation using AT-8 antibody for phosphorylated tau.

**Results:**

PBB5 showed specific binding to recombinant K18 tau fibrils by fluorescence assay, to post-mortem Alzheimer’s disease brain tissue homogenate by competitive binding against [^11^C]PBB3 and to tau deposits (AT-8 positive) in post-mortem corticobasal degeneration and progressive supranuclear palsy brains. Dose-dependent optoacoustic and fluorescence signal intensities were observed in the mouse brains following *i.v.* administration of different concentrations of PBB5. In vivo vMSOT brain imaging of P301L mice showed higher retention of PBB5 in the tau-laden cortex and hippocampus compared to wild-type mice, as confirmed by ex vivo vMSOT, epi-fluorescence, multiphoton microscopy, and immunofluorescence staining.

**Conclusions:**

We demonstrated non-invasive whole-brain imaging of tau in P301L mice with vMSOT system using PBB5 at a previously unachieved ~ 115 μm spatial resolution. This platform provides a new tool to study tau spreading and clearance in a tauopathy mouse model, foreseeable in monitoring tau targeting putative therapeutics.

**Supplementary Information:**

The online version contains supplementary material available at 10.1007/s00259-022-05708-w.

## Introduction

Abnormal cerebral deposition of pathological tau fibrils is a characteristic feature of tauopathy-related neurodegenerative diseases, including Alzheimer’s disease (AD), corticobasal degeneration (CBD), progressive supranuclear palsy (PSP), and parkinsonism linked to chromosome 17 [1]. The microtubule-associated protein tau (MAPT) is located intracellularly and is composed of six isoforms classified into 4-repeat (4R) and 3-repeat (3R) species [2]. Several tau positron emission tomography (PET) tracers have been developed, including first-generation [^18^F]flortaucipir, [^11^C]PBB3, and [^11^C]THK5351, [^18^F]THK5117 [3–7]; and second-generation [^18^F]MK-6240, [^18^F]PM-PBB3 (APN1607), [^18^F]JNJ-64326067, [^18^F]RO948, [^18^F]PI-2620, and [^18^F]GTP1 [8–13]. PET showed the spreading of tau in patients with AD, which correlates with axonal damage, neurodegeneration, functional network alterations, and cognitive impairment. Therefore, tau biodistribution represents a powerful biomarker with great potential in disease staging [14–23]. In addition, the tau tracer [^18^F]PM-PBB3 has been shown to facilitate the detection of distinct patterns in patients with PSP and CBD compared to AD, indicating its capability for differential diagnosis [9].

Transgenic mouse models (mutations in the *MAPT* gene) recapitulate pathological features of tauopathy and have greatly advanced our understanding of disease mechanisms [24–28]. Ex vivo high-resolution light-sheet microscopy with anti-tau antibodies or luminescent-conjugated oligothiophenes enabled whole-brain mapping of tau biodistribution and spread [29–31]. However, capturing early tau deposits in vivo is needed for a better understanding of the link with other pathological alterations in deep brain regions. In vivo PET imaging of cerebral tau accumulation in transgenic tauopathy mice has been achieved using [^18^F]PM-PBB3, [^11^C]PBB3, [^11^C]mPBB5, [^18^F]THK5117, [^18^F]JNJ-64349311, and 4R-tau specific tracers [^18^F]CBD-2115 [9, 32–40]. PET provides excellent accuracy to map the biodistribution of tau in human subjects. However, microPET has a limited spatial resolution (0.7–1.5 mm) relative to the small mouse brain, which hinders accurate detection of tau, especially in small subcortical brain regions [41]. Fluorescence tau imaging studies using PBB5, luminescent oligothiophene conjugated probes, BF-158, Q-tau 4, pTP-TFE, BODIPY derivative [36, 42–47], and fluorescently labeled antibodies [48] have been reported. However, fluorescence imaging provides a planar view and limited detection depth. Two-photon imaging of mice with a cranial window using HS-84, methoxy-X04, and fluorescently labeled antibodies [49–51] can follow the development of tau at cellular resolution but with a submillimeter field of view (FOV) and low penetration depth. Overall, existing imaging approaches are limited by either penetration depth or spatial resolution, which demands non-invasive imaging tools providing high-resolution performance at whole-brain scales.

Recently, volumetric multi-spectral optoacoustic tomography (vMSOT) imaging has been shown to provide previously unavailable capabilities to visualize the biodistribution of amyloid-β (Aβ) deposits in mouse models of AD amyloidosis [52–54]. vMSOT capitalizes on the high sensitivity of optical contrast and the high resolution provided by ultrasound [55, 56] and can attain a sufficient penetration depth to cover the whole mouse brain. State-of-the-art vMSOT embodiments enable whole-brain non-invasive imaging with ~ 115 µm spatial resolution [57–59], i.e., almost an order of magnitude finer than modern small-animal microPET scanners. In this study, we investigated on the capabilities of vMSOT assisted with the pyridinyl-butadienyl-benzothiazole derivative PBB5 probe to enable in vivo high-resolution 3D transcranial mapping of tau across the entire mouse brain in 4R-tau P301L mouse models [26]. The targeting performance of the PBB5 probe was further evaluated using post-mortem human brain tissues from patients with AD, PSP, and CBD.

## Methods

### Immunohistochemical staining of post-mortem brain tissues from patients with CBD and PSP

For fluorescence labeling with PBB5, deparaffinized sections were incubated in 50% ethanol containing 2 µM PBB5 at room temperature for 30 min. The samples were rinsed with 50% ethanol for 5 min, dipped into distilled water twice for 3 min, and mounted in nonfluorescent mounting media (VECTASHIELD; Vector Laboratories). Fluorescence images were captured using an FV-1000 confocal laser scanning microscope (Olympus, excitation at 635 nm and emission at 645–720 nm). Following fluorescence microscopy, all sections were autoclaved for antigen retrieval and immunohistochemically stained with AT-8-conjugated anti-phosphorylated tau antibodies (pSer202/pThr205, MN1020, Invitrogen, 1:250). Immunolabeling was then examined using a DM4000 microscope (Leica, Germany).

### In vitro [^11^C]PBB3 radiosynthesis and binding assay

Frozen tissues derived from the frontal cortex of an AD patient were homogenized in 50 mM Tris–HCl buffer, pH 7.4, containing protease inhibitor cocktail (cOmpleteTM, EDTA-free; Roche), and stored at − 80 °C until analyses. [^11^C]PBB3 was synthesized as described previously [7]. To assay radioligand binding with homologous or heterologous blockade, these homogenates (100 µg tissue) were incubated with 5 nM [^11^C]PBB3 (specific radioactivity, 86.9 GBq/µmol) in the absence or presence of nonradiolabeled PBB3 or PBB5 at varying concentrations ranging from 1 × 10^−11^ to 5 × 10^−7^ M in Tris–HCl buffer containing 10% ethanol, pH 7.4, for 30 min at room temperature. Non-specific binding of [^11^C]PBB3 was determined in the presence of 5 × 10^−7^ M PBB3. Samples were run in quadruplicate. The inhibition constant Ki was determined by using nonlinear regression to fit a concentration-binding plot to one-site and two-site binding models derived from the Cheng-Prusoff equation with GraphPad Prism version 9.0 (GraphPad Software), followed by the F test for model selection.

### In vitro fluorescence assay for the binding of probes to recombinant K18 tau fibrils

Detailed information on the probes and chemical compounds is listed in Suppl. Table 1 [60, 61]. Recombinant K18 4R tau was expressed and produced by *Escherichia coli* as described previously [62, 63] (Supplementary material). Details on recombinant K18 tau fibril production and characterization are in SFig. 1 and supplementary methods. The absorbance of the compounds was measured with a spectrofluorometer. Thioflavin T assays against K18 tau fibrils using a fluorometer (Fluoromax 4, Horiba Scientific, Japan) were performed as described previously [62], with two independent experiments and three technical replicates. PBB5 (excitation peak 630 nm, concentration 1.6 μM) was dissolved in Milli-Q H_2_O or dimethyl sulfoxide and further diluted in 1 × PBS (Gibco). PBB5 was then mixed with 5 μL of tau K18 fibril solution in a 45-µL quartz cuvette (quartz SUPRASIL Ultra Micro Cell, Hellma). The solution was incubated for 1 min at room temperature and resuspended, and fluorescence was measured with a spectrofluorometer using the corresponding excitation wavelength.

### Transmission electron microscopy

Then, 4 μL of the fibril samples (~ 50 μM) in PBS was applied directly to the negatively glow-discharged carbon-coated copper grids, followed by incubation for 1 min at room temperature. The excess solution was gently removed using Whatman filter paper. This step was followed by staining the samples with 10 μL of an aqueous phosphotungstic acid solution (1%, pH 7.2) for 1 min. The excess stain on the grid was then wiped off with filter paper, and the grid was washed with double-distilled water and air-dried. Finally, the images were recorded at ScopeM (ETH core facility) on an FEI Morgagni 268 electron microscope.

### Animal models

Transgenic *MAPT P301L* mice overexpressing human 2 N/4R tau under the neuron-specific Thy1.2 promoter (pR5 line, C57B6. Dg background) [26, 42, 64–66] and wild-type littermates were used (18 months old, *n* = 10 each group, both genders). For resolution characterization, one female athymic nude mouse (5 weeks old, JanvierLab, France) was used. Animals were housed in individually ventilated cages inside a temperature-controlled room under a 12-h dark/light cycle. Pelleted food (3437PXL15, CARGILL) and water were provided ad libitum. All experiments were performed in accordance with the Swiss Federal Act on Animal Protection.

### Post-mortem human brain tissues

Post-mortem human brains were obtained from autopsies carried out at the Center for Neurodegenerative Disease Research of the University of Pennsylvania Perelman School of Medicine on patients with AD, CBD, and PSP. Tissues for homogenate binding assays were frozen, and tissues for histochemical and immunohistochemical labeling were fixed in 10% neutral buffered formalin followed by embedding in paraffin blocks. All procedures involving the use of human materials were performed in accordance with the ethical guidelines of the Institutional Review Boards of the University of Pennsylvania and the National Institutes for Quantum and Radiological Science and Technology.

### In vivo imaging with the hybrid fluorescence and vMSOT system and resolution characterization

Simultaneous vMSOT and planar fluorescence imaging pre-, during, and post-*i.v.* bolus injection of PBB5 was performed using a previously established hybrid system consisting of an epi-fluorescence fiberscope and a vMSOT system capable of covering the entire mouse brain. The FOV is 10 × 10 mm^2^ for epi-fluorescence imaging and 15 × 15 × 15 mm^3^ for vMSOT, while the spatial resolution is approximately 40 μm and 115 μm for epi-fluorescence and vMSOT, respectively (Fig. 2) [53, 58, 67–71]. Mice were first anesthetized with an initial dose of 4% isoflurane (Abbott, Cham, Switzerland) in an oxygen/air mixture (200/800 mL/min) and subsequently maintained at 1.5% isoflurane in oxygen/air (100/400 mL/min) throughout the measurement. The fur and scalps over the head of the mice were then removed. The mice were placed in the prone position on a heating pad with feedback control to maintain a constant body temperature. The mice were subsequently injected with a 100 μL bolus containing PBB5 (Fig. 3, dissolved in dimethyl sulfoxide, 0.1 M PBS pH 7.4) through the tail vein. To establish the optimal dosage, four P301L and four wild-type mice were used for the dose response experiment (5, 25, 50 mg/kg weight). In the subsequent experiment, a dose of 25 mg/kg body weight was chosen and used in the following experiment. For vMSOT, the pulse repetition frequency of the laser was set to 25 Hz, and the laser wavelength was tuned between 550 and 660 nm (5 nm step) on a per pulse basis. Epi-fluorescence imaging was performed by coupling the same beam from the pulsed OPO laser into the excitation fiber bundle. The excited fluorescence field was collected by an imaging fiber bundle comprised of 100,000 fibers and then projected onto an EMCCD camera (Andor iXon life 888, Oxford Instruments, UK). vMSOT and epi-fluorescence signals were recorded simultaneously before injection (108 s duration), during injection (432 s duration with i.v. injection starting at 30 s after the beginning of acquisition) and 20, 40, 60, 90, and 120 min postinjection (108 s duration each). For resolution characterization, one female athymic nude mouse (*n* = 1, 5 weeks old, Janvier Lab, France) was imaged in vivo.

### vMSOT image reconstruction and multi-spectral analysis

During the experiments, vMSOT images were reconstructed in real time by using a graphics processing unit (GPU)-based implementation of a back-projection formula [52, 53, 72]. The reconstructed images were further processed offline to unmix the biodistribution of PBB5 [53]. Specifically, per-voxel least square fitting of the spectral signal profiles to a linear combination of the absorption spectra of oxygenated hemoglobin (HbO) and PBB5 was performed. Only wavelengths between 600 and 640 nm (10 nm step) were considered to avoid the strong changes in optical absorption and attenuation at shorter wavelengths and the laser instabilities at longer wavelengths. The absorption peak of PBB5 lies in this wavelength range, which is preferable for an optimal unmixing performance. The unmixing components (absorbing substances) considered were determined by comparing the unmixed biodistribution of the probe with that obtained by subtracting the preinjection image during injection of the probe. Generally, unmixing is performed by considering three components, namely the probe of interest (PPB5), HbO, and deoxygenated hemoglobin (HbR). However, it was found that including HbR as a component for unmixing led to larger errors in the biodistribution of the probe. The probe absorption spectra were experimentally determined as the average spectra of the differential (baseline-subtracted) vMSOT image during bolus perfusion at several major vessels in the brain. The vMSOT spectrum of PBB5 approximately matched the absorption spectrum measured with a spectrophotometer (Avantes BV, Apeldoorn, The Netherlands). The absorption spectrum of HbO was taken from an online database [73]. The effective attenuation coefficient was estimated by considering a constant reduced scattering coefficient of 10 cm^−1^ for all mice and an optical absorption coefficient corresponding to the unmixed biodistribution of blood and PBB5.

### Coregistration with MRI atlas and VOI analysis of the vMSOT data

Registration between vMSOT and MRI/atlas provides an anatomical reference for regional analysis [53, 74, 75]. These images were coregistered with T_2_-weighted structural MRI images (Ma-Benveniste-Mirrione-T_2_ [76]) in PMOD 4.2 (Bruker, Germany) by two readers independently. Volume-of-interest (VOI) analysis of 15 brain regions was performed using the embedded Mouse VOI atlas (Ma-Benveniste-Mirrione) in PMOD [53]. Specifically, the dynamic time course and retention (60 min) of regional PBB5 absorbance intensity (a.u.) were calculated. The extracranial background signal was removed with a mask from the VOI atlas.

### Ex vivo hybrid vMSOT and fluorescence imaging

To validate the in- and ex vivo signal, one P301L mouse was perfused under ketamine/xylazine/acepromazine maleate anesthesia (75/10/2 mg/kg body weight, *i.p.* bolus injection) with ice-cold 0.1 M PBS (pH 7.4) and 4% paraformaldehyde in 0.1 M PBS (pH 7.4), fixed for 4 h in 4% paraformaldehyde (pH 7.4), and then stored in 0.1 M PBS (pH 7.4) at 4 °C. The dissected brain was imaged using vMSOT and hybrid epifluorescence imaging. The brain was cut coronally using a mouse brain matrix (World Precision Medicine, USA) to a thickness of 2 mm at approximately − 2 to 0 mm and imaged again using the same setup. For this, the spherical array was positioned pointing upwards and filled with agar gel to guarantee acoustic coupling, which served as a solid platform to place the excised brain and brain slice. Uniform illumination of the brain surface was achieved by inserting three arms of the fiber bundle in the lateral apertures of the array and a fourth arm providing light delivery from the top. All recorded OA signals were normalized with the calibrated wavelength-dependent energy of the laser pulse. The biodistribution of the probe was estimated via multi-spectral unmixing considering the vortex component algorithm (VCA) considering optical wavelengths from 600 to 655 nm (5 nm step) [77, 78].

### Ex vivo multiphoton microscopy and immunofluorescence and confocal imaging

Fixed brains from one P301L mouse and one wild-type mouse were imaged at 20 × magnification using a Leica TCS SP8 Multiphoton microscope and analyzed using ImageJ (NIH, United States). Lambda scan 3D rendering identical setting resolution with Z stack and gain were used. Horizontal brain Sects. (40 μm) were cut and costained with PBB5 and anti-phosphorylated tau (pSer202/pThr205) antibody AT-8 (details in Suppl. Table 1). Sections were counterstained using 4’,6-diamidino-2-phenylindole DAPI [64]. The brain sections were imaged at × 20 magnification using an Axio Oberver Z1 and at × 63 magnification using a Leica SP8 confocal microscope (Leica, Germany) for co-localization of PBB5 with AT-8. The images were analyzed using ImageJ (NIH, USA).

### Statistics

Group comparison of PBB5 absorbance in multiple brain regions at different time points was performed by using two-way analysis of variance with Bonferroni post hoc analysis (GraphPad Prism, Switzerland). The difference in fluorescence at 60 min was compared using a two-tailed Student’s *t* test. All data are presented as mean ± standard deviation. Pearson’s rank correlation analysis was used to compare vMSOT and epifluorescence imaging data and reliability analysis. Significance was set at ∗ *p* < 0.05.

## Results

### vMSOT resolution characterization

**Fig. 1:**
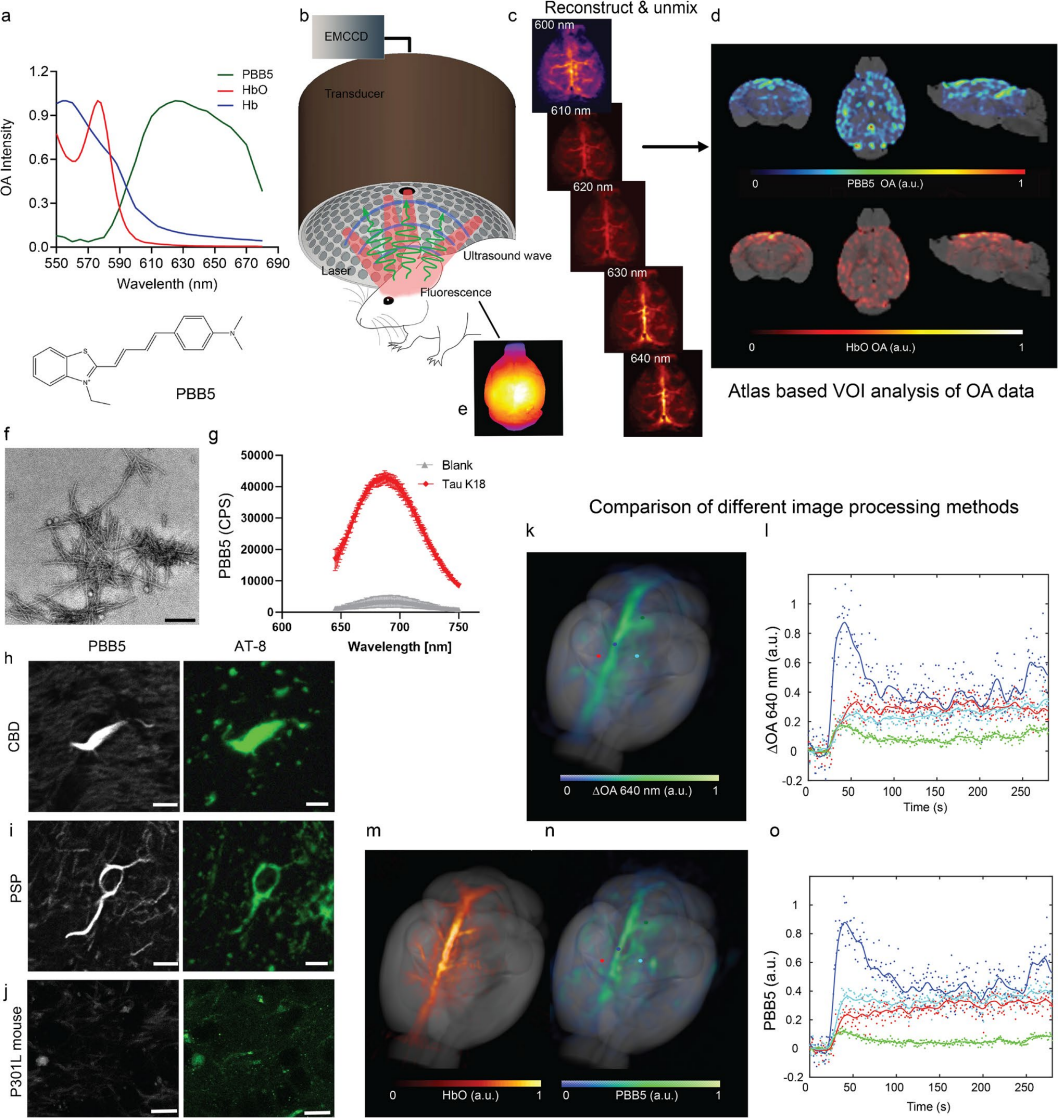
Non-invasive tau epifluorescence-vMSOT imaging pipeline: (**a**) chemical structure of the probe PBB5 and extinction spectrum of HbO and Hb along with the spectrum of PBB5 measured by volumetric multi-spectral optoacoustic tomography (vMSOT); (**b**) setup of the hybrid epifluorescence-vMSOT system for tau mapping across the entire mouse brain; (**c**) volumetric reconstructions of the in vivo vMSOT data for five distinct excitation wavelengths (600, 610, 620, 630, 640 nm) used for spectral unmixing; (**d**) coronal, horizontal, and sagittal views of PBB5 and HbO; absorbance intensity scale, 0–1. (**e**) Simultaneous epi-fluorescence imaging in one P301L mouse brain after *i.v*. injection of PBB5. (**f**–**j**) PBB5 characterization of recombinant fibrils and staining of the human brain. (**f**) Transmission electron microscopy image of the K18 tau fibril. scalebar = 200 nm. (**g**) Fluorescence binding assay using PBB5 on K18 4R tau fibrils and blank (dd. water) with PBB5, CPS, counts per sound; (**h**, **i**, **j**) PBB5-positive and AT-8-positive inclusions indicated coiled bodies in the caudate/putamen from patients with corticobasal degeneration (CBD) and motor cortex from progressive supranuclear palsy (PSP) and hippocampal sections from P301L mice; scalebar = 10 μm. AT-8, an anti-phosphorylated tau antibody. (**k**–**l**) Comparison of vMSOT processing methods. The baseline-subtracted single wavelength vMSOT 3D rendering image acquired at 640 nm (**k**) matches the multispectrally unmixed biodistribution of PBB5 (**n**). (**l**, **o**) Time-lapse curves of multispectrally unmixed PBB5 signals and baseline-subtracted 640 nm signals corresponding to selected points, cortex (red), superior sagittal sinus (dark blue), hippocampus (green), and vessel (light blue), indicated in (**k**) and (**n**). The multispectrally unmixed biodistribution of HbO reveals major cerebral vessels (**m**)

**Fig. 2:**
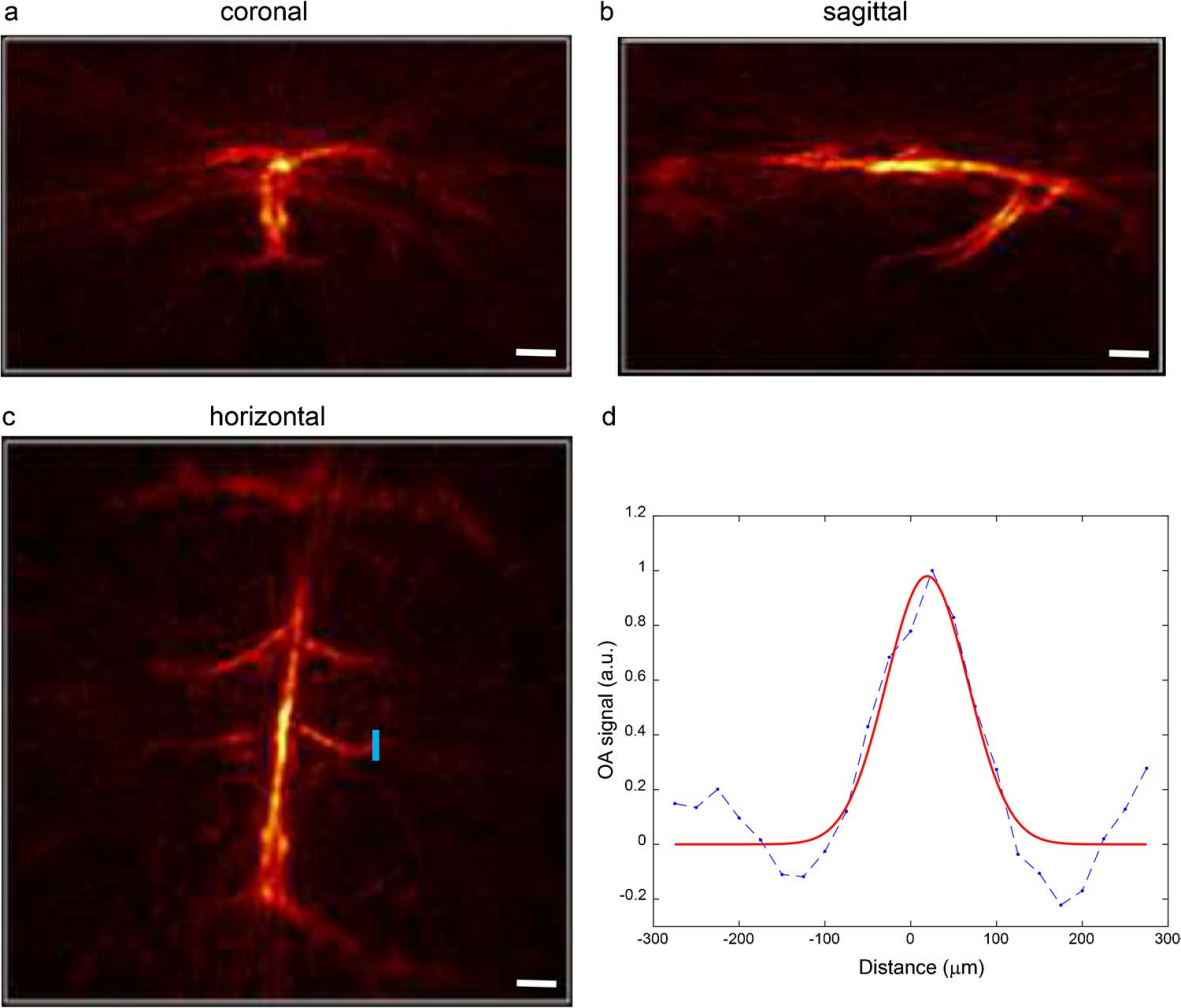
Characterization of the vMSOT resolution for transcranial imaging. (**a**–**c**) Maximum intensity projection (MIP) of the vMSOT image of the brain of a 6-week-old nude mouse (unmixed signal corresponding to oxygenated hemoglobin) through the intact scalp and skull in the coronal (**a**), sagittal (**b**), and horizontal (**c**) view; scale bar = 1 mm. (**d** One-dimensional vMSOT signal profile along the blue line indicated in the horizontal MIP image (**c**). The width of the fitted Gaussian curve is 115 μm. Blue line in d, raw profile; red line in d, fitted profile

We used a recently developed concurrent vMSOT and fluorescence imaging setup and data analysis pipeline for non-invasive transcranial 3D mouse brain imaging (Fig. 1b–d). First, we characterized the vMSOT resolution as the reconstructed size of the smallest cerebral vessels visible in an vMSOT image of a 5-month nude mouse brain recorded in vivo (Fig. 2). Specifically, the unmixed signal corresponding to HbO was considered as it provides the best vascular contrast for the smallest vessels. Note that the resolution is established by the detection bandwidth and angular coverage of the array of ultrasound sensors employed. Therefore, it does not depend on the absorbing substance generating the signal or on the depth from the tissue surface. The murine skull was also found not to play a role in resolution degradation for the mouse considered. The size of the selected vessel, located underneath the skull, was estimated as the full width at half maximum (FWHM) of the fitted Gaussian curve to the selected image profile.

### In vitro fluorescence binding assays in recombinant fibrils

We produced tau fibrils using bacterially produced recombinant monomers of the 4R-tau isoform called K18. The K18 tau fibrils were validated using the ThT assay (SFig. 1a), transmission electron microscopy (Fig. 1f), and western blot (SFig. 1b). To characterize the binding properties of PBB5 to tau fibrils and aggregates in vivo, we first studied the absorbance spectrum, affinity, binding kinetics, and specificity of PBB5 towards recombinant tau K18 fibrils (Fig. 1g).

### Staining in human brain

To investigate whether PBB5 binds to tau aggregates in the mammalian brain, we stained the caudate/putamen from patients with CBD and the motor cortex from PSP with both PBB5 and anti-phosphorylated tau antibody (AT-8). The latter was used as a positive control as it was shown to specifically bind tau inclusions in the brain [7]. Staining using PBB5 and anti-phosphorylated tau antibody (AT-8) in the caudate/putamen from patients with CBD and motor cortex from PSP showed an overlapping signal, which indicates that PBB5 is capable of recognizing AT-8-positive coiled bodies (Figs. 1h, i) and argyrophilic threads in oligodendrocytes (SFigs. 2a,b), and tufted astrocyte (SFig. 2c).

### Binding assays on human brain tissue

We further characterized the binding properties of PBB5 using brain tissues from patients with different tauopathies, including AD brain tissue with mixed 3R, 4R-tau, and CBD and PSP brain tissues with 4R-tau. Competitive binding assay in AD brain homogenates using different concentrations of unlabeled PBB5 and PBB3 against [^11^C]PBB3 (concentration 5 nM, specific activity 86.9 GBq/mmol, radiochemical purity 96.7%) indicated an inhibition constant (Ki) = 181.5 nM, and partial replacement for PBB5 (*R*^2^ = 0.9889, *n* = 4), compared to Ki = 2.5 nM for PBB3 (*R*^2^ = 0.9669, *n* = 4) (SFig. 2c).

### Non-invasive in vivo vMSOT of PBB5 uptake in the mouse brain

The absorption spectrum of PBB5 expands within the far-red range (~ 590–690 nm, Fig. 1a), where light penetration is significantly enhanced with respect to shorter wavelengths. This facilitates distinguishing the biodistribution of PBB5 from endogenous chromophores such as HbR and HbO via spectral unmixing of vMSOT images acquired in vivo. The surface-weighted PBB5 biodistribution was also measured in epi-fluorescence mode in both P301L and wild-type mice by means of a custom-built concurrent planar fluorescence-vMSOT system (Fig. 1b) as described in detail elsewhere [52, 53]. The vMSOT imaging data analysis pipeline consisted of the following steps. First, 3D vMSOT images were reconstructed for multiple excitation wavelengths (Fig. 1c). Then, spectral unmixing was performed to isolate the bio-distributions of HbO and PBB5. Finally, co-registration with a magnetic resonance imaging (MRI) mouse brain atlas [76] was performed for VOI analysis (Fig. 1d). After *i.v.* bolus injection of PBB5 in mice through the mouse tail vein (*n* = 20 in total), an increase in the fluorescence and/or spectrally unmixed PBB5 signal was observed in the mouse brain parenchyma, arguably indicating that the probe passed the blood–brain barrier. Epi-fluorescence images of the brain corroborated the increase in signal associated with PBB5, albeit providing no depth information and significantly inferior resolution compared to vMSOT (Fig. 1e).

### Spectral unmixing of the vMSOT data

Spectral unmixing can generally isolate the biodistribution of any spectrally distinctive probe from endogenous absorbers in biological tissues, preferably with a peak absorption wavelength lying in the wavelength range of interest. However, spectral coloring effects associated with wavelength-dependent attenuation of light lead to cross-talk artifacts when considering the theoretical spectra of the absorbing substances present in the sample [79, 80]. This is particularly important for spectral windows exhibiting sharp variations in hemoglobin absorption, e.g., at wavelengths of approximately 570–600 nm (Fig. 1a) [73]. The wavelengths and absorbing components were optimized so that the unmixed biodistribution of PBB5 matches that obtained by subtracting a reference image taken before injection for the sequence vMSOT images taken at 640 nm wavelength (see the “vMSOT image reconstruction and multi-spectral analysis” section). We found that the unmixing performance was optimal when considering five wavelengths (600, 610, 620, 630, and 640 nm) and only HbO and PBB5 as absorbing components by comparing the averaged unmixed images with the averaged differential (baseline-subtracted) vMSOT images at 640 nm for 3–7 min after injection of the probe (Figs. 1k–o). This corroborates the validity of multispectral unmixing with the selected wavelengths and components as a method to isolate the biodistribution of PBB5. Note that the images shown in Figs. 1k and n correspond to time points when PBB5 is still circulating in blood vessels, i.e., the unmixed biodistribution of PBB5 is expected to partially match the unmixed biodistribution of HbO.

### Dosage-dependent performance

**Fig. 3:**
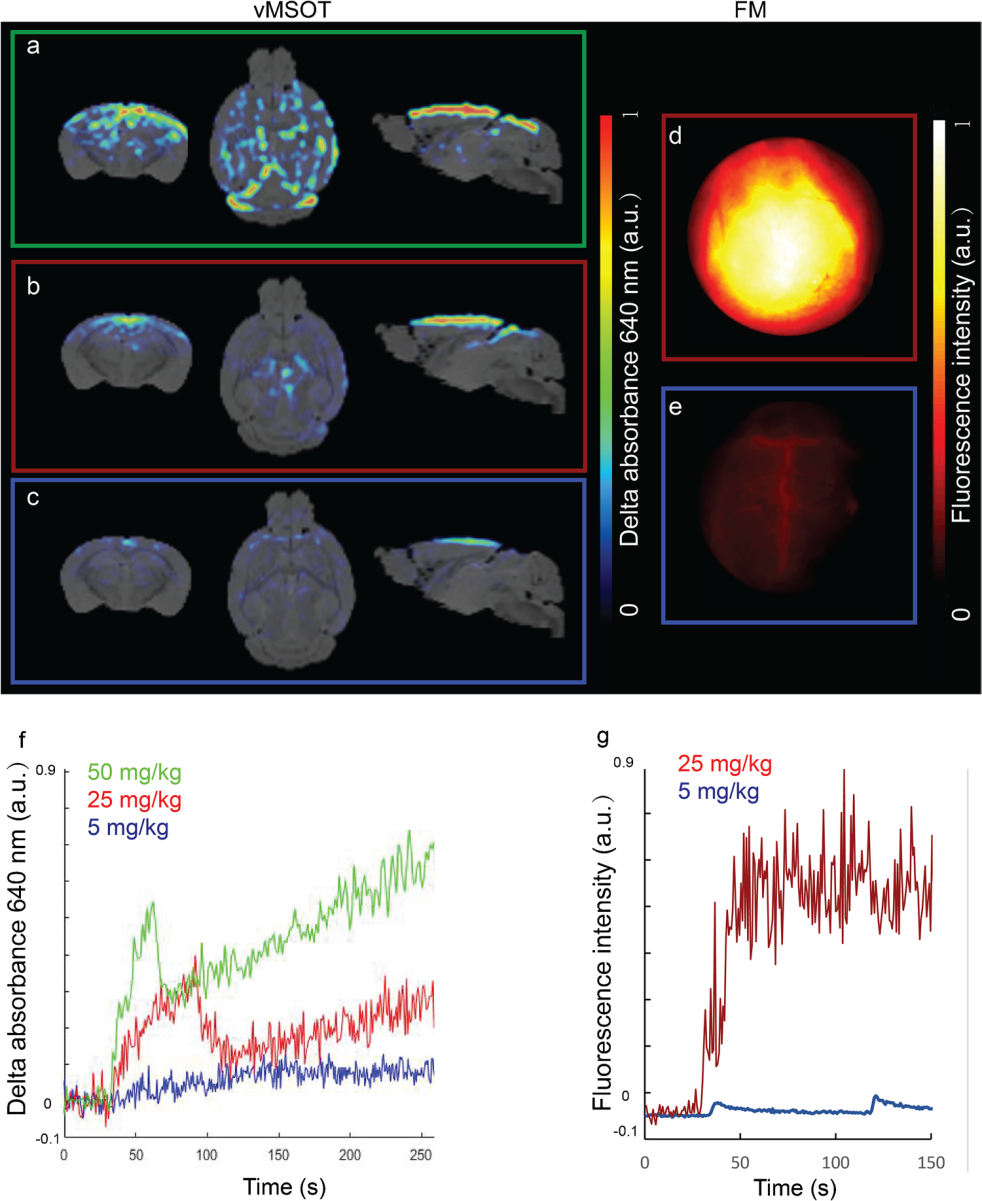
Dose determination for in vivo tau imaging with vMSOT. (**a**–**e**) vMSOT images of three different concentrations of PBB5, 5 mg/kg weight (blue square), 25 mg/kg weight (red square; b) and 50 mg/kg weight (green square, a) and epifluorescence images of 5 mg/kg weight (blue square, e), 25 mg/kg weight (red square, d); (f) Time curve of unmixed PBB5 absorbance profile during the first 300 s (within 7-min dynamic *i.v.* injection using three different concentrations of PBB5, 5 mg/kg weight (blue line), 25 mg/kg weight (red line), and 50 mg/kg weight (green line). No clear signal increase was detected using a 5 mg/kg weight dose. (**g**) Fluorescence intensity curve of PBB5 using 5 mg/kg weight (light blue) and 25 mg/kg weight (dark blue). PBB5 was injected *i.v*. at 30 s

The optimal dosage of PBB5 to allow clear signal detection in the vMSOT images was established by testing different concentrations of PBB5 (5, 25, 50 mg/kg weight) in P301L and wild-type mice (*n* = 2–3 each group at each concentration). A dependence on the unmixed PBB5 signal in the vMSOT images with the concentration of the probe was clearly observed at 20–60 min post injection (Figs. 3a–c, f). Due to the abundant endogenous hemoglobin signal in the mouse brain, a negligible signal increase was detected using 5 mg/kg PBB5. A total of 25 mg/kg PBB5 (*i.v*.) provided a sufficient vMSOT signal increase to be detected in the unmixed images. Fluorescence imaging results indicated a dose-dependent signal similar to that of PBB5 vMSOT imaging; i.e., a very intense signal was observed at 25 mg/kg PBB5, while a sufficient fluorescence signal increase was also observed using 5 mg/kg PBB5 (Figs. 3d, e, g).

### PBB5 biodistribution in P301L and wild-type mice

**Fig. 4:**
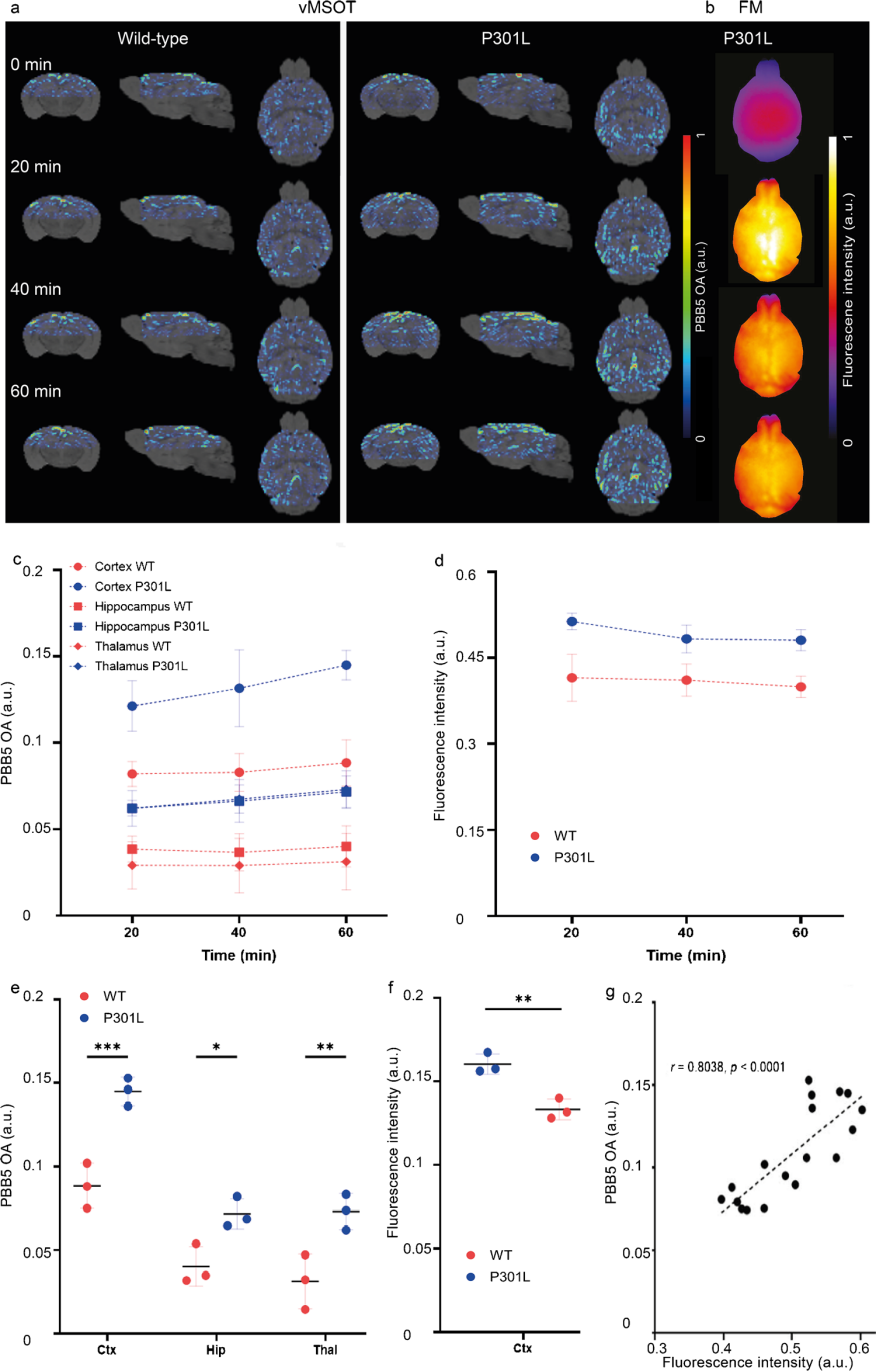
Regional tau distribution revealed by in vivo vMSOT imaging using PBB5 probe in P301L and wild-type mice, (**a**) wild-type (WT) and transgenic P301L mice; at pre-injection, 20, 40, and 60 min following dye administration showing coronal, sagittal, and horizontal views overlaid over the masked magnetic resonance imaging-based brain atlas. PBB5 absorbance signal strength is indicated by rainbow color-map; (**b**) example of epi-fluorescence images from one P301L mouse at 20, 40, and 60 min following dye administration; (**c**, **d**) time course of cortical, hippocampal, thalamic volume-of-interest PBB5 signal (absorbance signal), and cortical region-of-interest fluorescence intensity; (**e**, **f**) regional comparison of probe absorbance signal retention and fluorescence intensity at 60 min post-injection; Data are presented as the mean ± SD; P301L (*n* = 3), and NTL (*n* = 3); *p < 0.05, **p < 0.01, ***p < 0.001 comparison between WT and P301L mice. Cortex, Ctx; Hippocampus, Hip; Thalamus, TH; (**h**) correlation between optoacoustic and fluorescence imaging across different mice using Pearson rank analysis

**Fig. 5:**
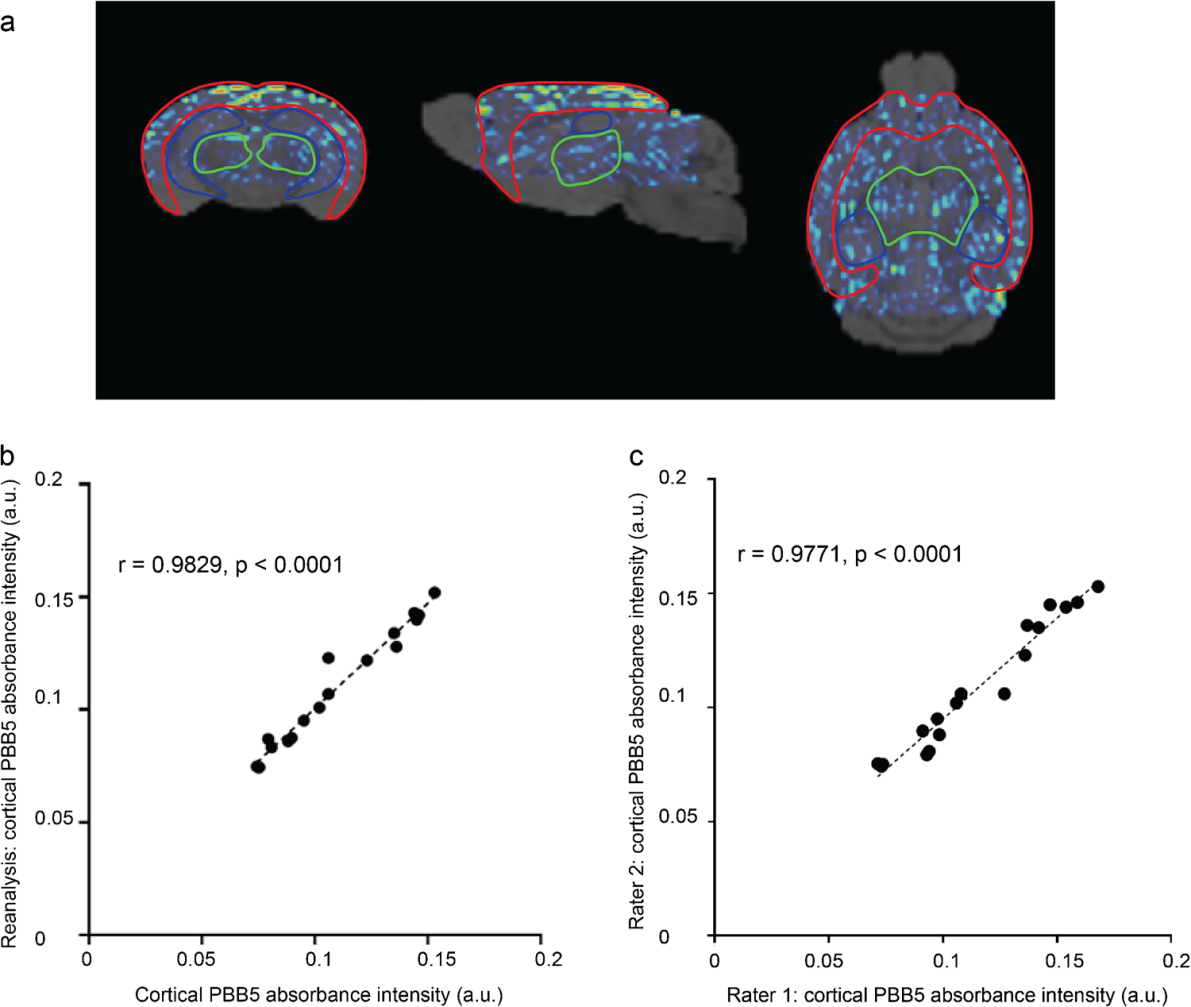
Reliability of volume-of-interest (VOI) analysis. **a** VOI labeling of the segmented brain areas—cortex, red; thalamus, green; hippocampus, blue. **b** Intra-rater reliability. **c** Inter-rater reliability. Analysis and reanalysis using PMOD volume-of-interest analysis process. Pearson rank analysis indicated a robust correlation between two independent analyses for the cortical PBB5 absorbance intensity (a.u.)

P301L (*n* = 3) and wild-type mice (*n* = 3) were imaged at different time points before, during, and after injection of PBB5 (25 mg/kg weight *i.v.*) using the vMSOT system. The unmixed images for the PBB5 channel were superimposed onto the MRI atlas for VOI analysis (Fig. 4a, SFig. 3). The time courses of PBB5 (absolute OA (a.u.)) in different brain regions of P301L and wild-type mice were assessed (Fig. 4c). Significantly higher PBB5 OA at 60 min post-injection was observed in the cortex, hippocampus, and thalamus of P301L mice than in wild-type mice (Fig. 4e, SVideo 1,2). Similar temporal profiles of vMSOT and planar fluorescence signals were observed throughout the cortical region (Fig. 4d, SFig. 4). A robust correlation was observed between the fluorescence and unmixed vMSOT PBB5 absorbance signal (*p* < 0.0001, Pearson’s rank correlation analysis) (Fig. 4g, Fig. 5a–c). The test–retest correlation analysis between independent analyses is shown in Fig. 5, indicating the repeatability of the VOI analysis (interrater and intrarater reliability).

### Ex vivo validation of PBB5 detection of tau in P301L mouse brains

**Fig. 6:**
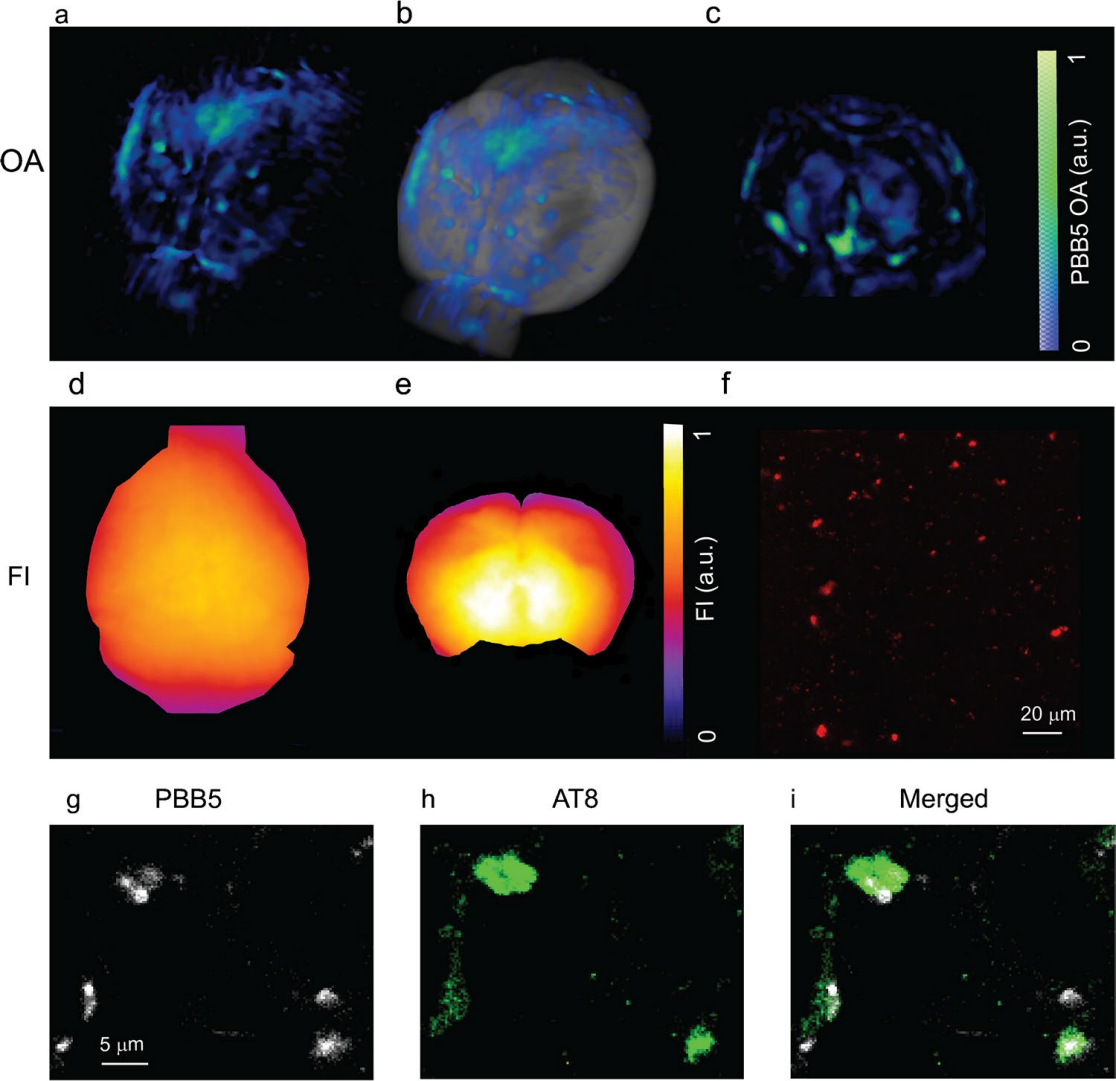
Ex vivo validation using vMSOT, epi-fluorescence imaging, multiphoton microscopy, and immunofluorescence staining. (**a**–**c**) Ex vivo vMSOT of whole brain and brain slices at 90 min after PBB5 *i.v.* injection; (**a**) 3D rendering of ex vivo vMSOT data unmixed for PBB5 distribution in P301L mouse brain; (**b**) overlay of a on MRI structural data; (**c**) ex vivo vMSOT of 1 mm mouse brain slice data unmixed for PBB5 distribution in P301L mouse brain. The PBB5 absorbance signal strength is indicated by a blue-green color map; (**d**, **e**) epi-fluorescence of a and c; (**f**) multiphoton microscopy (MPM) regional quantification multiphoton. Scale bar = 20 μm; (**g**–**i**) confocal microscopic images of hippocampal sections from P301L mice. PBB5 (white) and Alexa488-AT-8 (green) in the hippocampal areas. Scale bar = 5 μm

To validate the in vivo imaging results, the mouse brains were dissected after in vivo imaging and imaged ex vivo using the same vMSOT setup. The accumulation of PBB5 signals in the cortex and hippocampus of P301L mice suggests specific binding of the probe to these regions known to express high tau load. Ex vivo PBB5 epifluorescence images corroborated the tau accumulation in vMSOT, although it was not possible to resolve different regions (Figs. 6a, b, d). Imaging on coronal brain slices (~ 2 mm thickness, coronal slices cut using a brain matrix at Bregma − 2–0 mm) indicates retention of signal in the brain of P301L mouse (Figs. 6c, e). To further validate the in vivo PBB5 signal distribution imaged with vMSOT with higher resolution, we imaged fixed brains from P301L and wild-type mice by multiphoton microscopy. Consistent with the in vivo imaging findings, tau deposit morphology was clearly observed in tissue slices, with stronger PBB5 signals found in the cortex and hippocampus of P301L mice (Fig. 6f). Immunofluorescence staining performed on horizontal brain tissue sections from P301L and wild-type mice costained with anti-phosphorylated tau AT-8 antibody further confirmed the detection of PBB5 in tau (Fig. 1j, Figs. 6g–i).

## Discussion

New tools for non-invasive mapping of tau deposits with high resolution in animal models of tauopathy are imperative for understanding the accumulation and spreading of tau deposits [81] and for translational development of tau-targeted therapeutic and diagnostic tools [82, 83]. Herein, we identified PBB5 as a suitable tau imaging probe for vMSOT that binds with high sensitivity and specificity to tau aggregates in vitro and in vivo. This was used to establish a novel in vivo transcranial vMSOT imaging approach to map whole brain tau deposits at ~ 115 μm resolution in a P301L mouse model.

The criteria for selecting an appropriate tau-specific probe for vMSOT imaging include a suitable absorption spectrum to allow unambiguous unmixing from the endogenous signal of blood (preferably with peak absorption at > 600 nm optical wavelength), high affinity, low toxicity, low non-specific binding, photostability, low toxicity, low molecular weight, and suitable lipophilicity to allow sufficient blood–brain barrier passage and biocompatibility [84]. Herein, we chose PBB5 for its peak absorption at 630–640 nm (where the absorption of hemoglobin decays), which facilitates distinguishing it from blood. A competitive binding assay against [^11^C]PBB3 and PBB5 was further shown to have an affinity Ki of 181 nM in post-mortem cortical tissue from patients with AD. The binding affinity is in line with the previously reported affinity of PBB5 [36]. Although the specificity and brain penetration of PBB5 is lower than that of PBB3 (with peak absorption at 405 nm) [36] or PM-PBB3 (emission at 525 nm) [9], its near-infrared (NIR) absorption spectrum allows for epi-fluorescence and vMSOT imaging of deep brain regions. Staining with PBB5 and AT-8 of brain tissues from caudate/putamen patients with CBD and motor cortex from PSP showed an overlapping signal demonstrating that PBB5 is capable of recognizing tau accumulation in coiled body and argyrophilic threads inside oligodendrocytes in brain from CBD and PSP, as well as tufted astrocytes in brain from PSP.

Tau plays an important role in the pathogenesis of AD and other primary tauopathy diseases, such as CBD and PSP [29, 85, 86]. Ongoing clinical trials targeting tau reduction have shown promising results. These include antibodies gosuranemab BIIB092 or non-pharmacological treatments [87–91]. Tau imaging has, however, been challenging due to the structural diversity of tau isoforms, the difference between 4 and 3R-tau, its intracellular location, and the specificity and off-target binding of tau imaging probes [92, 93]. PET assisted with the tau tracer [^18^F]PM-PBB3 has been shown to detect different patterns in patients with PSP and CBD compared to AD, indicating a role in differential diagnosis [9]. Recent cryogenic electron microscopy has shown that PM-PBB3 binds to tau fibrils in the AD brain [94]. An in silico study reported THK5351 probes, T807 binding to different sites on tau fibrils [95] and off-target binding sites [93]. Previous autoradiography and PET studies indicated that PBB analogs THK5351 or THK5117 and JNJ-64349311 but not T807 can detect tauopathy in tau mouse models (P301L, PS19 line) [32, 34–37, 96, 97].

In P301L (CaMKII) mice, tau deposits start at 5 months of age, first in the limbic system (entorhinal cortex and hippocampus) and subsequently spreading to the neocortex [26, 98]. Tauopathy deposits in P301L (Thy1.2) mice [26] are most pronounced in the cortex, amygdala, and hippocampus, moderate in the brain stem and striatum, and negligible in the cerebellum. Thus, we chose cerebellum as reference brain region. Similar to PBB3 and PM-PBB3, PBB5 detects AT-8-stained neurofibrillary tangles, ghost tangles, tau deposits in astrocytes, and oligodendrocytes in the brain from PSP and CBD [7]. In P301L as well as in other tauopathy mouse models, the neurofibrillary tangle is rear, and less fibrillar structure is present in the mouse brain [26, 37, 98]. The cortical and hippocampal signals detected by vMSOT in vivo and ex vivo using PBB5 are in accordance with immunofluorescence staining results and with the known tau distribution in the P301L mouse brain [64, 98]. NIR fluorescence imaging using PBB5 and PET using [^11^C]mPBB5 have been previously reported for mapping tau deposition in the brain stem and spinal cord of P301S mice [36]. However, NIR fluorescence imaging detection in deep brain regions was hindered by strong absorption and scattering of the excitation light and emitted fluorescence. Submillimeter-scale intravital microscopy enables the visualization of tau deposits but is highly invasive and can only cover a very limited FOV [50]. We recently reported on large FOV fluorescence microscopy imaging of tau in P301L mice with 6 micron resolution, which only provided a planar view [42]. As the spatial resolution of vMSOT is not altered by photon scattering but rather governed by ultrasound diffraction, it enables high-resolution mapping and quantification of endogenous tissue chromophores or spectrally distinctive exogenous probes at millimeter- to centimeter-scale depths [55, 67, 99].

There are several limitations in the current study that need to be highlighted. We did not take into account the spectral coloring effect associated with wavelength-dependent optical attenuation, which may cause distortion in the vMSOT spectra rendered from deep locations [80, 100]. These factors may lead to cross-talk artifacts in the unmixed images corresponding to the contrast agent. Advanced algorithms are required to attain more accurate performance [100]. A reference tissue model for kinetic models will be potentially useful for improved quantification. In addition, future longitudinal studies are required to determine the sensitivity and specificity of the proposed methodology, how early PBB5-positive tau can be detected, and whether it can follow the spreading of tau in the brain [101].

## Conclusions

We demonstrated non-invasive whole-brain imaging of tau in P301L mice with a state-of-the-art vMSOT system at ~ 115 μm spatial resolution, which is not feasible with other imaging modalities. This platform provides a new tool to study tau spreading and clearance in a tauopathy mouse model, foreseeable in monitoring tau-targeting therapeutics.

## Supplementary Information

Below is the link to the electronic supplementary material.

Supplementary file1 (DOCX 1602 KB)

Supplementary file2 (AVI 428 KB)

Supplementary file3 (AVI 626 KB)

## Data Availability

The datasets generated and/or analyzed during the current study are available in the repository zenodo 10.5281/zenodo.4699067.

## Code availability

Upon request.

## Declarations

### Preprint information

This manuscript has been previously deposited in a preprint server [102, 103].
