# Supplementary material for: Non-invasive imaging of tau-targeted probe uptake by whole brain multi-spectral optoacoustic tomography
Source: Eur J Nucl Med Mol Imaging. 2022 Feb 7;49(7):2137–52. doi: 10.1007/s00259-022-05708-w (PMC9165274; doi:10.1007/s00259-022-05708-w)
Supplement: Supplementary file 1 — Supplementary file1 (DOCX 1602 KB) [file 259_2022_5708_MOESM1_ESM.docx]

**Supplementary materials**

**Supplementary Figure 1**. K18 tau fibril characterization.

**Supplementary Figure 2.** PBB5 characterization and staining of the human brain.

**Supplementary Figure 3**. *In vivo* tau imaging with vMSOT.

**Supplementary Figure 4.** Epi-fluorescence imaging using PBB5 (i.v.) in P301L and wild-type mice

**Supplementary Video 1.** Noninvasive *in vivo* vMSOT of PBB5 probe distribution inP301L mouse brains at 60 minutes post-*i.v.* injection. vMSOT signals were unmixed for PBB5 (blue–green) and Hb/HbO (red–yellow, vascular signal component)

**Supplementary Video 2.** Noninvasive *in vivo* vMSOT imaging of PBB5 probe distribution inP301L mouse brains at 60 minutes post-i.v. injection. vMSOT signals were unmixed for PBB5 (blue–green) and Hb/HbO (red–yellow, vascular signal component) and overlaid with MRI brain atlas shown in grayscale,

**Supplementary Table 1:** List of primary antibodies and compounds in western blot and immunohistochemistry

**Supplementary Methods**

Recombinant K18 tau fibril production and characterization


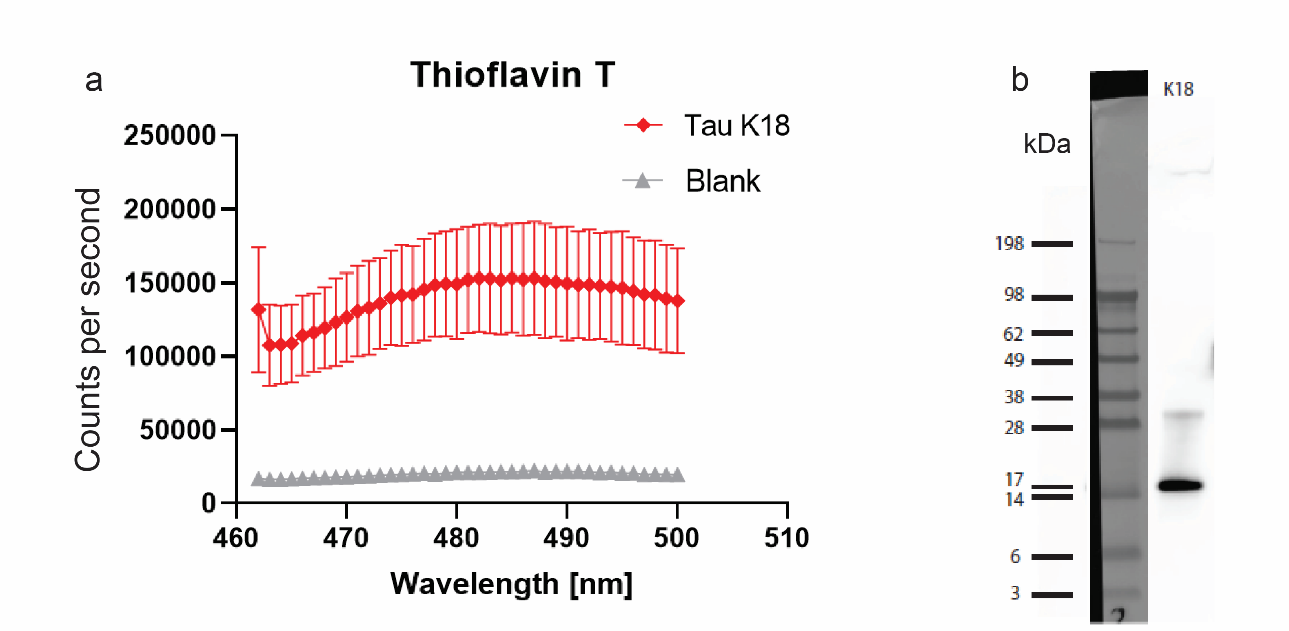


**Supplementary Fig 1**. **K18 tau Fibril characterization.** (**a**) Thioflavin T assay on K18 4 repeat tau fibril and blank (dd. water) using spectrofluorometric measurements; (**b**) Western blot using anti-Tau (RD4) antibody clone 1E1/A6 indicated the K18 tau monomer.


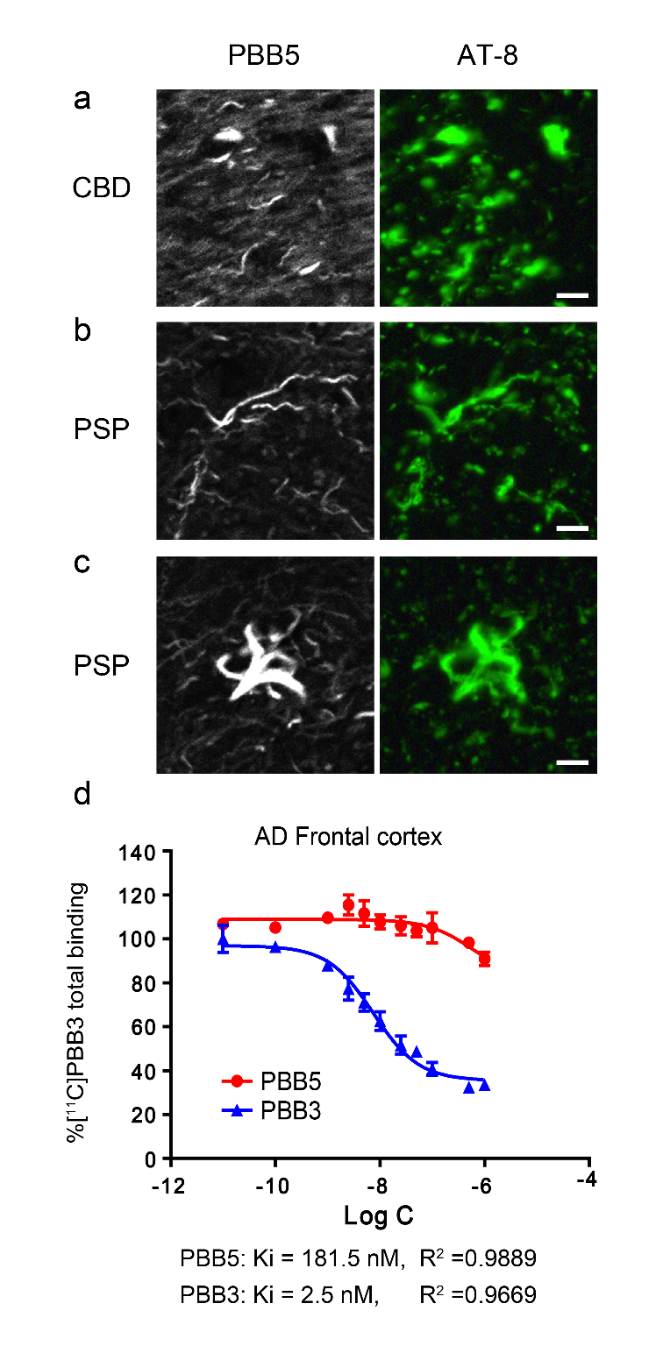


**Supplementary Fig. 2 PBB5 characterization and staining of the human brain.** (**a-c**) PBB5-positive and AT-8-positive inclusions indicated argyrophilic threads (**a, b**) accumulated in oligodendrocytes and tufted astrocytes (**c**) in the caudate/putamen from patients with corticobasal degeneration (CBD) and motor cortex from progressive supranuclear palsy (PSP); scale bar = 10 μm. AT-8: an anti-phosphorylated tau antibody. (**d**) Binding of [^11^C]PBB3 in cortical homogenates derived from Alzheimer’s disease (AD). Total (specific + non-specific) binding of 5 nM [^11^C]PBB3 in an AD temporal cortex sample blocked homologously by unlabeled PBB3 (blue) and heterologously by unlabeled PBB-5 (red). Inhibition of radioligand binding was described by a 1-site model, and parameters resulting from curve fits are indicated.


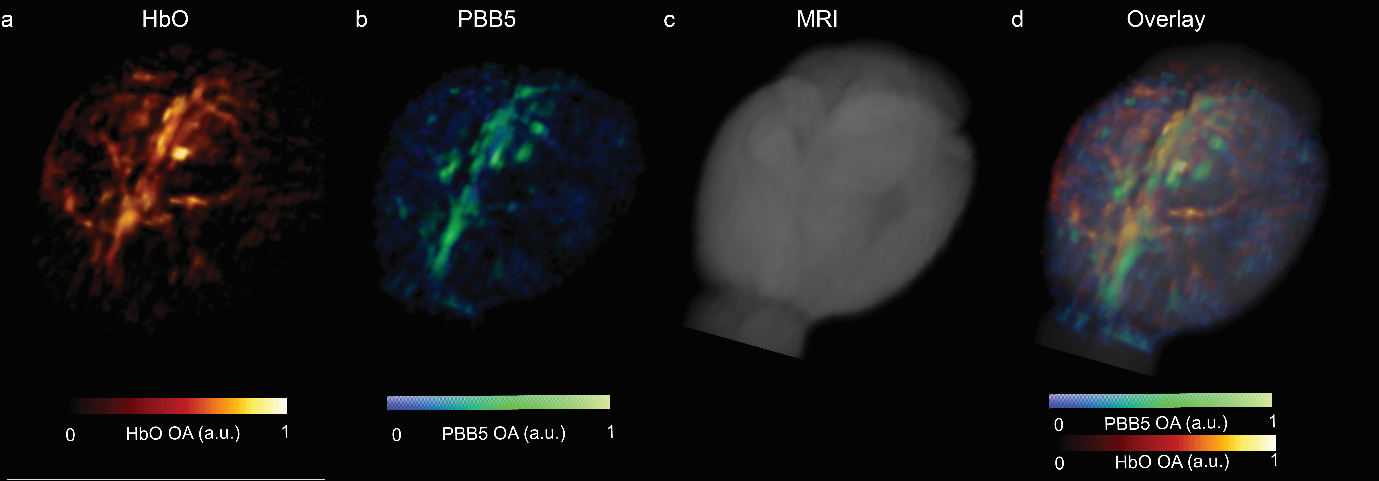


**Supplementary Fig 3**. ***In vivo* tau imaging with vMSOT.** 3D rendering of vMSOT data in one P301L mouse brain at 60 minutes post-PBB5 i.v. injection. (**a)** Image acquired at 600 nm excitation wavelength reveals major cerebral vessels. (**b**) vMSOT image unmixed for PBB5 distribution, (**c**) MRI structural data. (**d**) Overlay of the vMSOT images shown in (a) and (b) and the MRI image shown in (c).


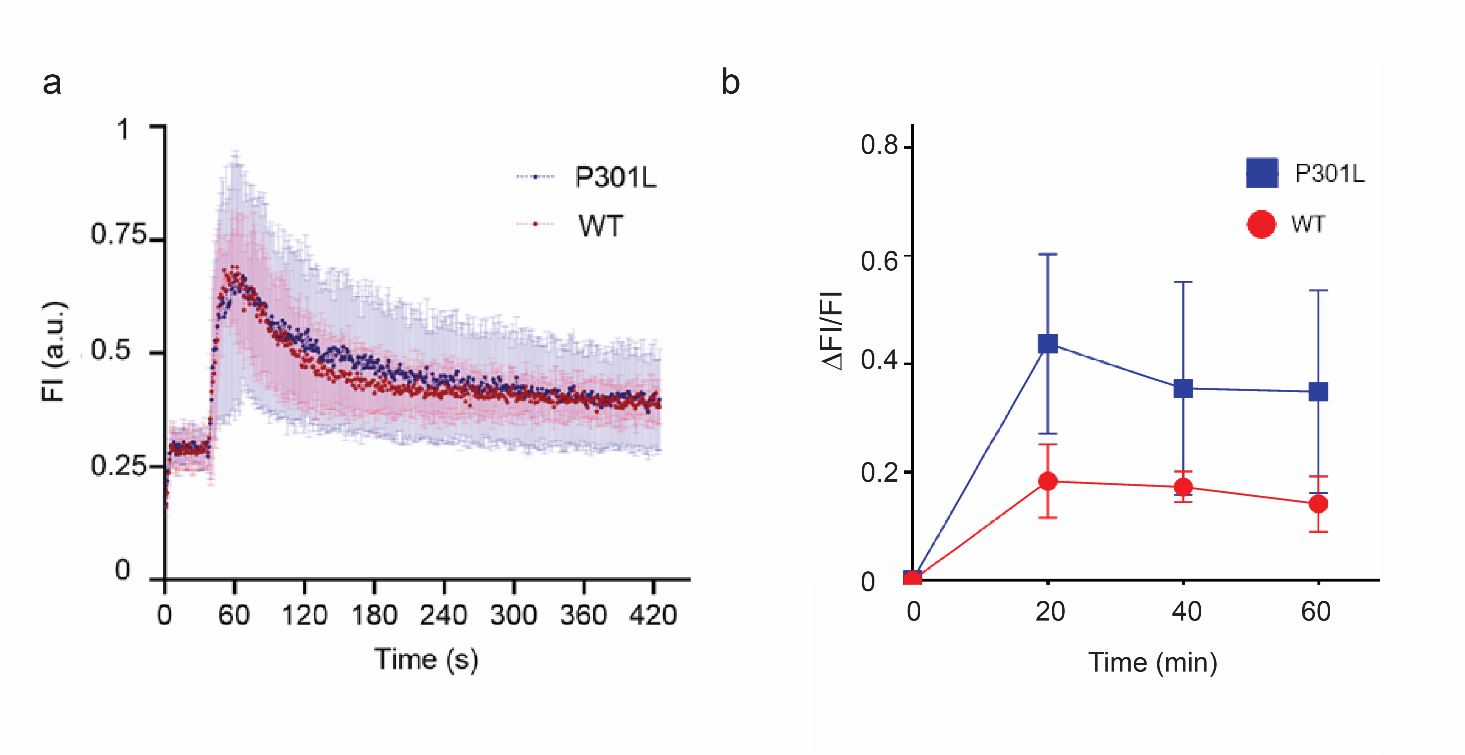


**Supplementary Fig 4. Epi-fluorescence imaging using PBB5 (i.v.) in P301L and wild-type mice** (**a**) Characterization of fluorescence intensity (FI) during perfusion after i.v. injection of 25 mg/kg weight PBB5 using dynamic vMSOT imaging in the brains of P301L (n=3) and wild-type mice (n=3). (**b**) ΔFI/FI in P301L (n=3) and wild-type mice (n=3) over 60 minutes after i.v. injection PBB5 25 mg/kg weight.

**Supplementary Table 1:** List of primary antibodies and compounds in western blot and immunohistochemistry

| **Antibody/Compounds** | **Company** | **Cat. No.** | **Dilution** |
| --- | --- | --- | --- |
| DAPI | Sigma | D9542-10MG | 1:1000 |
| Goat-anti-Rabbit Alexa488 | Invitrogen | A11034 | 1:200 |
| AT-8 | Invitrogen | MN1020 | 1:250 (human);  1:1000 (mouse) |
| PBB5 | RadiantDye |  | 50 μM |
| Normal goat Serum | Sigma |  |  |
| Anti-Tau (RD4) Antibody, clone 1E1/A6 | Sigma | 05-804 | 1:1000 |
| Anti-mouse-IgG antibody conjugated to Horseradish Peroxidase | Jackson ImmunoResearch | 115-035-166 | 1:5000 |
| Pierce™ ECL Western Blotting Substrate | ThermoFisher Scientific | 32106 |  |
| VECTASHIELD fluorescent mounting media | Vector Laboratories | H-1000-10 |  |

**Supplementary Methods**

**Recombinant K18 tau fibril production and characterization**

Recombinant K18 4R tau was expressed and produced by Escherichia coli (*E. coli*) as described previously [1, 2].

Transformation and culture: competent *E. coli* cells (strain BL21 DE3*) were transformed with the bacterial expression vector pRK172 containing the genomic sequence encoding the 4 repeat microtubular binding region (MTBR) of tau (kindly provided by the Marc Diamond lab). For transformation, we used the standard heat shock method; frozen chemically competent cells were thawed on ice, and 2 μL of the Tau K18 expression vector was added to the cells, which were incubated on ice for 30 minutes. Heat shock was performed at 42°C for 90 seconds, and the transformed cells were incubated on ice for 4 minutes. One milliliter of sterile LB medium was added to the cells, which were then incubated under agitation for 1 hour (37°C, 400 rpm). The solution was centrifuged (1000 × g, 2 minutes), and 700 µl of the supernatant was discarded. The pellet was resuspended in the remaining supernatant (≈ 300 µl), spread onto an agar plate supplemented with ampicillin (10 µg/mL) and incubated overnight (37°C). The next morning, a single colony was picked with a sterile toothpick and incubated in 200 mL of sterile LB medium and ampicillin under agitation (120 rpm, 37°C) overnight. Twenty milliliters of the preculture was transferred into 2 liters of sterile LB medium (containing ampicillin) and incubated under agitation (120 rpm, 37°C). A total of 6 liters of LB was prepared for tau K18 expression. The optical density at 590 nm (OD590) was measured repeatedly. When the OD590 reached 0.8, 1 mL of 1 M isopropyl β-D-1-thiogalactopyranoside (IPTG) per liter of growth medium was added, and the incubation was continued overnight. On the next day, the solution was centrifuged (3000 × g, 15 mins, 4°C), and the pellet was collected and stored at -20°C.

Extraction and Purification: The pellet was recovered from the freezer (-20°C), thawed on ice and resuspended in BRB80 buffer with 0.1% β-mercaptoethanol (BME) and 1 mM phenylmethylsulfonyl fluoride (PMSF). Cell lysis was performed by sonication and subsequently letting the suspension flow through a microfluidizer. The cells were sonicated three times in 50 mL aliquots at 50% power (Bandelin UW 2070 ultrasonic homogenizer), and each round lasted 30 seconds. The probe of the sonicator was cooled down after three intervals to prevent overheating of the sample. The subsequent disruption of the bacteria (Microfluidics, M-110S) was performed at a pressure of 40 psi for two rounds. Afterwards, the suspension was centrifuged (3000 × g, 20 mins, 4°C), and the supernatant was recovered. The cell lysates were then cleared by a boiling step that consisted of incubating the supernatant for 10 minutes in a water bath set at 100C. After boiling, the samples were incubated at room temperature for 1-2 h until they reached 25-30°C. Using this procedure, heat-sensitive proteins are irreversibly denatured and therefore retained in the pellet when a centrifugation (3000 × g, 4°C, 15 min) step is subsequently applied to the sample. The supernatant was filtered through a 0.22 µm polyethersulfone (PES) membrane (Steritop Filter, Millipore Express PLUS) and then stored on ice.

Cation exchange chromatography: Cation exchange chromatography was performed using a phosphocellulose column: 3 g of cellulose phosphate (Sigma Aldrich (discontinued), CAS: 9015-14-9) was washed with NaOH (0.5 M), sodium phosphate (0.5 M, pH 7), HCl (0.5 M) and twice as much sodium phosphate (0.5 M, pH 7). The washed cellulose phosphate mesh was poured into a borosilicate glass chromatography column (Sigma, 18 mL, Luer-Lock). The column was packed and equilibrated by letting 100 mL of BRB80 buffer containing 0.1% BME flow through. Chromatography was performed at 4°C with a flow rate of 0.3 mL/min (pump: Pharmacia Biotech P-50). The column was subsequently eluted with BRB80 + 0.1% BME with increasing volumes of NaCl: 30 mL of 0.2 M NaCl in BRB80 + BME, 30 mL of 0.4 M NaCl in BRB80 + BME, 30 mL of 0.6 M NaCl in BRB80 + BME and 30 mL of 1 M NaCl in BRB80 + BME. 10 mL fractions were collected and analysed by SDS–PAGE to identify the fractions containing the protein of interest. For SDS–PAGE, aliquots from each elution fraction, the flow-through, and the solution before chromatography were mixed with Laemmli sample buffer and subsequently boiled at 95°C for 5 minutes. The gel (NuPAGE 4-12% Bis-Tris, ThermoFisher Scientific) was loaded with 15 µL of sample per well and 2 µL of Protein Standard (BioRad Precision Plus Protein Dual Color Standard) and run at a constant voltage of 160 V. The gel was stained with a Coomassie-based stain (InstantBlue, expedeon) (and stored in H_2_O for later on performed western blot).

Dialysis and Lyophilization: Based on the SDS–PAGE readout, the fractions containing the highest amount of protein (corresponding to the fractions showing the most prominent bands at approximately 15 kDa) were selected and pooled. The protein solution was then dialysed (3 kDa cut-off membrane, Spectrum Spectra/Por) against Milli-Q H2O at 4°C for three days. Every 12 hours, the water in the dialysis container was exchanged. After dialysis, the protein solution was collected in Falcon tubes, frozen in liquid nitrogen, and finally lyophilized (Alpha 2-4 LD, Christ). Long-term storage of the lyophilized samples was conducted at -80°C.

Fibrillization: To induce fibrillization, the lyophilized proteins were dissolved in PBS buffer pH 7.4 (Gibco) containing 0.05% NaN3. The proteins were resuspended in 200 μl of PBS/NaN3, and several aliquots were prepared in 1.5 ml Eppendorf tubes. For fibrilization, the protein solutions were incubated at 37°C under agitation (500-700 RPM) in an Eppendorf thermomixer.

Thioflavin T (ThT) assay: After incubation of the protein solutions, fibrillization was verified by Thiaflovin T fluorescence assay: 45 μL of 5 μM ThT was mixed with 5 μL of Tau K18 in a 45 µL quartz cuvette (quartz SUPRASIL Ultra Micro Cell, Hellma). The mixtures were incubated for 1 minute and resuspended in a pipette, and the fluorescence intensity in the spectrum of 460-500 nm was measured with a spectrofluorometer (FluoroMax-4, Horiba Jobin Yvon) using an excitation wavelength of 450 nm.

Western blot: The gel (Novex 10-20%, Tricine, Thermo Fisher Scientific) was loaded with 10 μg of Tau K18. The samples were mixed with Laemmli sample buffer and boiled at 95°C for 5 minutes. SDS–PAGE was performed at 100 V for 1 h 45 min. The gel was transferred to a nitrocellulose membrane (part of Invitrogen iBlot Transfer Stack, nitrocellulose, Thermo Fisher Scientific) using a dry blotting system (Invitrogen iBlot 2, Thermo Fisher Scientific). The membrane was washed with 1× PBS and 0.1% Tween 20 (PBST) and blocked with 5% fat-free milk. Tau K18 was detected using a monoclonal Tau antibody (anti-Tau 4-repeat isoform RD4, clone 1E1/A6, Sigma Aldrich 05-804). The primary antibodies were diluted in 5% fat-free milk (anti-Tau 1:1,000). As a secondary antibody, a purified anti-mouse IgG antibody conjugated to horseradish peroxidase was used (Jackson ImmunoResearch, 115-035-166), which was diluted 1:5,000 in 5% fat-free milk. The membranes were incubated with the primary antibodies at 4°C overnight. The membranes were washed with PBST and subsequently incubated with secondary antibodies at room temperature for 2 hours and then washed with PBST. To detect Tau K18, ECL Prime Western Blotting Detection Reagents (Cytiva, RPN2232) were used. Pictures were taken with ImageQuant LAS 4000 (GE Healthcare).

**Reference**

1. Burmann BM, Gerez JA, Matecko-Burmann I, Campioni S, Kumari P, Ghosh D, Mazur A, Aspholm EE, Sulskis D, Wawrzyniuk M *et al*: **Regulation of alpha-synuclein by chaperones in mammalian cells**. *Nature* 2019.

2. Gerez JA, Prymaczok NC, Rockenstein E, Herrmann US, Schwarz P, Adame A, Enchev RI, Courtheoux T, Boersema PJ, Riek R *et al*: **A cullin-RING ubiquitin ligase targets exogenous alpha-synuclein and inhibits Lewy body-like pathology**. *Sci Transl Med* 2019, **11**(495).
